# Soil Phosphorus Forms and Profile Distributions in the Tidal River Network Region in the Yellow River Delta Estuary

**DOI:** 10.1155/2014/912083

**Published:** 2014-05-20

**Authors:** Junbao Yu, Fanzhu Qu, Huifeng Wu, Ling Meng, Siyao Du, Baohua Xie

**Affiliations:** ^1^Key Laboratory of Coastal Environmental Processes and Ecological Remediation, Yantai Institute of Coastal Zone Research (YIC), Chinese Academy of Sciences (CAS), Shandong Provincial Key Laboratory of Coastal Environmental Processes, YICCAS, Yantai, Shandong 264003, China; ^2^University of Chinese Academy of Sciences, Beijing 100049, China; ^3^College of Environmental Science and Engineering, Ocean University of China, Qingdao, Shandong 266100, China

## Abstract

Modified Hedley fraction method was used to study the forms and profile distribution in the tidal river network region subjected to rapid deposition and hydrologic disturbance in the Yellow River Delta (YRD) estuary, eastern China. The results showed that the total P (P_t_) ranged from 612.1 to 657.8 mg kg^−1^. Dilute HCl extractable inorganic P (P_i_) was the predominant form in all profiles, both as absolute values and as a percentage of total extracted P_i_. The NaOH extractable organic P (P_o_) was the predominant form of total extracted P_o_, while Bicarb-P_i_ and C.HCl-P_o_ were the lowest fractions of total extracted P_i_ and P_o_ in all the P forms. The Resin-P concentrations were high in the top soil layer and decreased with depth. The Pearson correlation matrix indicated that Resin-P, Bicarb-P_i_, NaOH-P_i_, and C.HCl-P_i_ were strongly positively correlated with salinity, TOC, Ca, Al, and Fe but negatively correlated with pH. The significant correlation of any studied form of organic P (Bicarb-P_o_, NaOH-P_o_, and C.HCl-P_o_) with geochemical properties were not observed in the study. Duncan multiple-range test indicated that the P forms and distribution heterogeneity in the profiles could be attributed to the influences of vegetation cover and hydrologic disturbance.

## 1. Introduction

Phosphorus (P), as a limiting nutrient, is one of the major plant nutrient elements second in importance to nitrogen (N) in terms of nutrient requirements for increased plant biomass production in ecosystem [1]. In its biogeochemical cycle, P is typically eroded from upland sources and transported, along with the sediment to which it is attached, to a final receiving water body [2, 3]. Before reaching the ocean, overland flow and runoff may travel through most of tidal wetlands, which are complex, dynamic, and continually changing due to the action of wind, currents, tides, and waves. Phosphorus in tidal river network region typically exists in many complex chemical forms, while investigations of P biogeochemistry often partition the pool of P into its labile and refractory components, especially with regard to soil P availability for plant or phytoplankton growth [4].

Meanwhile, most methods for available P determination attempt to quantify P solubility using different extractions, but few of those were related to P supply rates and relevant to plant uptake [5, 6]. To overcome this limitation, Hedley et al. [7] developed a method to extract P using a series of successively stronger reagents. At each step of the fractionation scheme, extracts could be assigned a role that could be used to characterize a chemical form of P. The sequential fractionation, in which the pool of soil P is partitioned into inorganic, organic, and microbial forms [8], can differentiate the plant-available forms (Resin-P_i_, Bicarb-P_i_, and Bicarb-P_o_) and refractory forms (NaOH-P_i_, NaOH-P_o_, D.HCl-P_i_, C.HCl-P_i_, C.HCl-P_o_, and Residual-P). Many previous studies have used the sequential fractionation scheme developed by Hedley, which was successfully used to separate forms of organically bound soil phosphorus from the geochemically bound fractions [9], to successfully describe the contribution of biological processes to the concentrations and disposition of P pools across a gradient of mineral weathering and soil development [10]. Among the studies reviewed, there are rare systematic studies of sequentially extractable P fractions in the tidal river network soils reported and no concerned documentation of the relationship between P forms of tidal wetlands and their chemical soil properties is available, especially for the soils in the tidal river network region of the Yellow River Delta (YRD) estuary in the east part of China.

The Yellow River, whose basin was the birthplace of ancient Chinese civilization, is one of the essential rivers for China's very existence. The YRD is the youngest natural coastal wetland ecosystem and the most intensive land-ocean interaction region among the large river deltas in the world [11]. The typical characteristics of the YRD are rapid deposit and fast evolution because the sediment load delivered into the sea accounts for 6% of the global rivers' sediment load into the sea [12]. Thus the Yellow River is regarded as the largest contributor of fluvial sediment load compared to the oceans in the world. The net increase of delta shoreline length was ~61.64 km with annual increase of ~1.81 km, and the net extension of area was ~309.81 km^2^ with rate of ~9.11 km^2 ^yr^−1^ within 34 years (1976–2009) [13].

The ability of wetlands to act as sinks for certain chemicals, sediments, and nutrients has been one of the main motivating factors for wetland protection. For the past few years, there are many studies focusing on the landscape pattern [14–16], biodiversity conservation [16], ecological restoration [17], and wetland evolution [11, 18] of the YRD. Unfortunately, few studies focused on the soil P forms and the relation of P distribution with vegetation cover and hydrologic disturbance in coastal areas [19–22]. In the present study, soil phosphorus forms and profile distributions in the tidal river network region were studied. Our objectives were (a) to fill a void of information on P forms of the tidal river network region soils, (b) to investigate the differences of contents and distribution of P forms of soils in river and tidal creek bed and bank by the sequential extraction procedure according to a modified Hedley fraction, (c) to correlate the content of P forms with basic chemical soil properties, and (d) to establish general differences in P forms and availability influence by vegetation cover and hydrologic disturbance.

## 2. Material and Methods

### 2.1. Study Area

**Figure 1:**
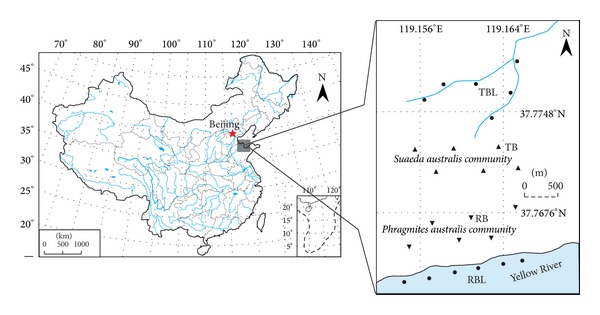
The tidal river network region in the Yellow River Delta estuary and sampling plots in wetlands (including bare river bed (BRB), river bank (RB), tidal bank (TB), and tidal creek bed (TCB)).

The studied tidal river network region (Figure 1) is located in the Yellow River Delta Natural Reserve (37°35′–38°12′N, 118°33′–119°20′E) established in 1992 by the State Council of China, which is between the Bohai Gulf and the Laizhou Bay in eastern China. As a wetland type reserve, which is well protected for the important habitat, breeding, or stopover place for the birds in China, it holds the most extensive, integrated, and youngest wetland ecosystem in the warm temperate zone of China. Due to deposition of lots of sand and mud carried by water of the Yellow River from the Loess Plateau, the main soil is typical saline alluvial soil (Fluvisols, FAO). The natural vegetation is salt-tolerant plants and aquatic plants. The predominant species in the tidal river network region are* Phragmites australis* and* Suaeda heteroptera *Kitag. This region is subjected to warm temperate continental monsoon climate with distinctive seasons and rainy summer. Average annual sunshine hours are 2590–2830 h, average annual temperature is 11.7–12.8°C, and the frost-free period is about 196 d. The average annual precipitation is 530–630 mm with nearly 70% of the precipitation falling mainly in summer, while the evaporation is 1900–2400 mm and the drought index is up to 3.56 [17].

### 2.2. Soil Collection

In order to examine the forms and distribution of soil phosphorus in the tidal river network region in the YRD estuary, along the Yellow River and a tidal creek, four soil sampling plots were collected for study on 27 April in 2011. The plots were demonstrated as follows: (1) bare river bed (BRB), which was in the Yellow River bed with no vegetation cover; (2) river bank (RB), which was on the river bank with* Phragmites australis* as dominated species cover; (3) tidal bank (TB), which was on the tidal creek with* Suaeda heteroptera *Kitag. as dominated species cover; and (4) tidal creek bed (TCB), which was in the tidal creek bed with no vegetation cover. In each plot, 6 replicate soil samples were collected using a stainless-steel slide hammer with an inner diameter of 3.5 cm. Each collected core was sectioned at 10 cm interval of 0–60 cm depth. The soil samples were stored immediately in polyethylene plastic bags after collection in the field. All soil samples were air-dried, grounded using a mortar and pestle, and then sieved 0.850 mm and 0.150 mm sieves prior to laboratory analysis.

### 2.3. Laboratory Analyses and Statistical Methods

A 0.5000 g soil sample was placed in a 50 mL plastic centrifuge tube with 30 mL of deionized water and the freely exchangeable fractions were removed by anion exchange resin in the chloride form. The plant available and moderately labile pool was extracted with NaHCO_3_ (0.5 M, pH = 8.5) and 0.1 M NaOH, whereas 1 M HCl removed the Ca-associated portion, since Fe- or Al-associated P that might remain unextracted after the NaOH extraction is insoluble in acid. Hot concentrated HCl was used to separate organic P (P_o_) from inorganic P (P_i_) in stable residual fractions. The residue left after the hot concentrated HCl extraction is unlikely to contain anything but highly recalcitrant P_i_ pool which can be digested with concentrated H_2_SO_4_ and H_2_O_2_ at 360°C. To determine total P in 0.5 M NaHCO_3_, 0.1 M NaOH, and concentrated HCl, extracts of dissolved organic matter were oxidized with ammonium persulfate before P analysis. A volume of 5 mL of solution was injected into a 50 mL volumetric flask. 0.5 g ammonium persulfate and 10 mL 0.9 M H_2_SO_4_ were weighed into the flask containing 5 mL of the NaHCO_3_ and NaOH extracts. The extracts were then autoclaved for 60 min (NaHCO_3_ and concentrated HCl) and 90 min (NaOH). Inorganic P in the 0.5 M NaHCO_3_ and 0.1 M NaOH extracts was determined by acidifying 10 mL aliquot in a 50 mL centrifuge tube with 6 mL and 1.6 mL of 0.9 M H_2_SO_4_ at 0°C for 30 min to precipitate OM. The OM was removed by carefully decanting the supernatant after centrifuging at 16000 rpm for 10 min. Organic P was estimated as the difference between total P and P_i_. The P concentration in the supernatant was determined by a Tu-1810 spectrophotometer (PERSEE, China), using the ascorbic acid molybdenum blue method described by Murphy and Riley [23]. The P fractionation procedure has been described in detail by Tiessen and Moir [24].

To analyze for pH, soil moisture (SM), soil salinity, and total organic carbon (TOC), the representative samples of dried and sieved soil were delivered to Yantai Institute of Coastal Zone Research, Chinese Academy of Sciences. Soil pH was measured with a Beckman pH meter with combination electrode (soil : water ratio = 1 : 5) and salinity was quantified with a conductivity bridge. Cutting ring and oven dry method was used to measure SM. TOC was measured using a LECO CN2000 combustion gas analyzer (AOAC International 1997). Al, Fe, and Ca in acid digested extract were measured by ICPS-7500 (Manufactured by Shimadzu, Japan).

The chemical analyses were performed at the Key Laboratory of Wetlands Ecology and Environment, Yantai Institute of Coastal Zone Research, Chinese Academy of Sciences. The P forms against concentrations of basic chemical properties and Duncan multiple-range test about P factions in the four plots were conducted using the Pearson correlation method. Correlation analyses and Duncan multiple-range test were conducted with SPSS 18.0 (SPSS, Inc. 2010).

## 3. Results and Discussion

### 3.1. General Characteristics of the Soils in the Tidal River Network Region

The mean pH of the tidal river network region soils which varied from 8.7 to 9.2 was strongly alkaline. The mean proportion of SM which ranged from 20.7 to 25.1% was high water content and the mean salinity which ranged from 0.4 to 22.2% was mainly hypersaline. Meanwhile, SM and salinity exhibited increasing gradients from the Yellow River bed to a tidal creek bed, which was primarily caused by the regionally geological, geochemical, and hydrologic conditions. The content of TOC ranged from 0.3 to 1.3% (Table 1), while the contents of Ca, Al, and Fe ranged 3.7–6.2%, 5.6–7.6%, and 2.4–4.1%, respectively (Table 1). Related research has also found that the soil characteristics in this region have low nutrients and high salinity [25]. These soils were characterized with low organic matter. Because the sediment to which they were attached in the YRD came from Loess Plateau by long transportation via the Yellow River, the organic matter was lost during the long transportation. These tidal river network region soils in the YRD estuary are classified as young hydromorphic alluvia (Fluvents) formed on fluvo-marine deposits. As a newborn estuarine coastal wetland, it was characterized by poor soil development with high soil salinity and low nutrient availability and only covered by salt-tolerant plant communities.

### 3.2. Distribution of Soil Phosphorus Fractions

Previous studies about the phosphorus fractions in an acid soil continuously fertilized with mineral and organic fertilizers revealed that the potential information about the distribution of soil P pools could help us to well understand the sinks and sources of P in the soil [26]. Related study has evaluated the mean total P fractions ranging from 471.1 to 694.9 mg kg^−1^ in the newly formed wetland soils in the Yellow River Delta [20]. In these soils in the tidal river network region, the mean content of total P (P_t_) was ranked as TCB (mean, 657.8 mg kg^−1^) > RB (mean, 649.2 mg kg^−1^) > TB (mean, 636.3 mg kg^−1^) ≫ BRB (mean, 612.1 mg kg^−1^) (Table 2). The P_t_ concentrations were larger in the top and bottom layers than those in the middle layers in all the soil profiles with an exception of BRB. The minimum value of P_t_ occurred in 20–30 cm layer in RB and TB, 30–40 cm layer in TCB, but 50–60 cm layer in BRB (Table 3 and Figure 3). Duncan multiple-range test indicated a marked difference among TCB, TB, and BRB, while the differences between TCB and RB, TB and RB were not significant (Table 2). The mean content of inorganic P forms with Resin-P, Bicarb-P_i_, NaOH-P_i_, D.HCl-P_i_, C.HCl-P_i_, and Residual-P (recalcitrant P_i_) ranged 9.6–16.6, 4.2–7.1, 4.0–6.2, 414.6–423.5, 45.0–78.0, and 36.9–47.1 mg kg^−1^, respectively (Table 2) while the mean content of organic P forms with Bicarb-P_o_, NaOH-P_o_, and C.HCl-P_o_ ranged 30.0–40.8, 22.1–43.9, and 22.0–30.1 mg kg^−1^, respectively (Table 2). In all the soils, total inorganic P was the predominant part and accounted for 82.7–90.9%.

**Figure 2:**
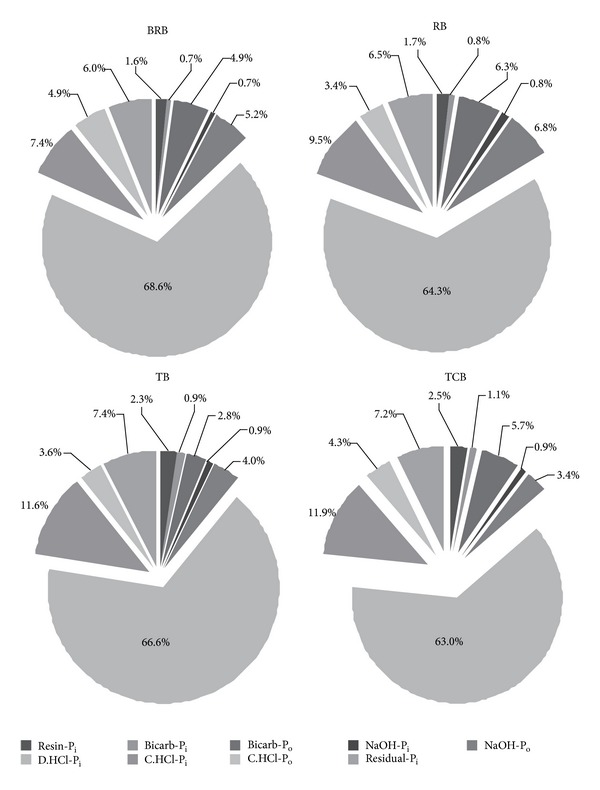
Soil P fractions in BRB, RB, TB, and TCB soils (P fractions including Resin-P, Bicarb-P_i_, NaOH-P_i_, D.HCl-P_i_, C.HCl-P_i_, Bicarb-P_o_, NaOH-P_o_, C.HCl-P_o_, and Residual-P).

**Figure 3:**
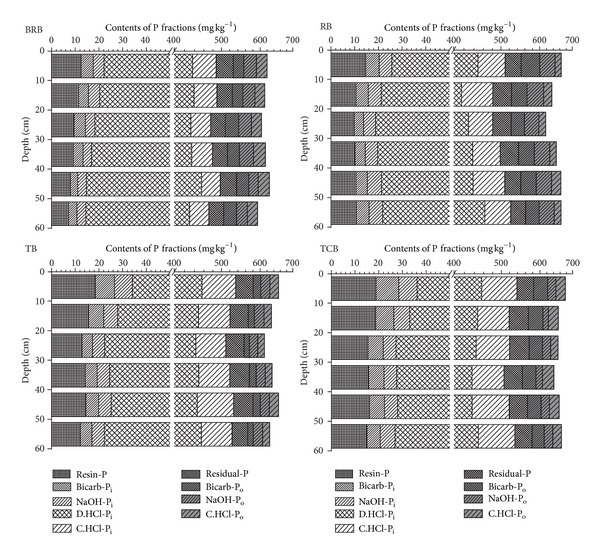
P fractions distribution in BRB, RB, TB, and TCB soil profiles (P forms including Resin-P, Bicarb-P_i_, NaOH-P_i_, D.HCl-P_i_, C.HCl-P_i_, Bicarb-P_o_, NaOH-P_o_, C.HCl-P_o_, and Residual-P).

The plant available P fractions—Resin P and Bicarb-P_i_/P_o_—summed to 7.2% of total P in the BRB and 8.8% in the RB, meanwhile 6.0% of total P in the TB and 9.3% in the TB (Table 2). Resin-P is reasonably well defined as freely exchangeable P_i_, since the resin extract does not chemically modify the soil solution. The mean content of Resin-P was ranked as TCB (16.6 mg kg^−1^, 2.5%) ≫ TB (14.6 mg kg^−1^, 2.3%) ≫ RB (11.0 mg kg^−1^, 1.7%) > BRB (9.6 mg kg^−1^, 1.6%) (Table 2 and Figure 2). In all profiles, the Resin-P concentrations were high in the top soil layer and decreased with depth (Table 3 and Figure 3). The most likely contributor to the effect of exchanging on Resin-P in the tidal river network region was salinity, which was an important feature of coastal wetlands that can affect P sorption.

Bicarbonate extracts a P_i_ fraction, which is likely to be plant available, since the chemical changes introduced are minor and somewhat representative of root action respiration. Bicarb-P_o_, which is easily mineralisable, is also likely to represent similar pools. The mean content of Bicarb-P_i_ which was ranked as TCB (7.1 mg kg^−1^ g, 1.1%) ≫ TB (5.8 mg kg^−1^, 0.9%) > RB (4.9 mg kg^−1^, 0.8%) > BRB (4.2 mg kg^−1^, 0.7%) (Table 2 and Figure 2) was the lowest fraction of total extracted P_i_. The mean contents of Bicarb-P_o_ of TB, BRB, TCB, and RB were 17.7 mg kg^−1^, 30.0 mg kg^−1^, 37.6 mg kg^−1^, and 40.8 mg kg^−1^, respectively, in soil profiles.* Suaeda heteroptera *Kitag., as pioneer plant community in TB in newly from coastal wetland, significantly enhanced the easily mineralisable P_o_ and increased P availability.

NaOH extractable P (NaOH-P_i_ + NaOH-P_o_) in the soil profiles averaged 36.0 mg kg^−1^ in BRB soils, 49.3 mg kg^−1^ in RB soils, 31.4 mg kg^−1^ in TB soils, and 28.36 mg kg^−1^ in TCB soils (Table 2). NaOH-P_o_ (mean: 3.4–6.8% of P_t_) was the predominant form of total extracted P_o_ (Figure 2). The depth distribution of Bicarb-P_i_ and NaOH-P_i_ in BRB, TB, and TCB was large in top soil and decreased with depth. The distributions in RB profiles showed that the low concentration of Bicarb-P_i_ was encountered at 20–30 cm depth and the high concentration of NaOH-P_i_ was encountered at 50–60 cm depth of the soil (Table 3 and Figure 3). Meanwhile, we did not observe vertical variation trends of Bicarb-P_o_ and NaOH-P_o_ and marked differences in the same profile were not shown.

Dilute HCl extractable inorganic P (D.HCl-P_i_) (mean: 63.0–68.6% of P_t_) was the predominant form in all profiles, both as absolute values and as a percentage of total extracted P_i_. Dilute HCl extractable P was clearly defined as inorganic P associated with Ca [5]. There were no significant vertical differences with respect to the soil D.HCl-P_i_ profiles in all the four profiles (Figure 3). The D.HCl-P_i_ is the predominant form (mean: 394.1–441.6 mg kg^−1^; 63.0%–68.6%), both as absolute values and as a percentage of total extracted P, which suggests that a relatively high proportion of inorganic P is in no-directly plant available forms. The depth distribution of D.HCl-P_i_ in all profiles showed little differences in top soil and dramatically increased in bottom soil. The maximum content of D.HCl-P_i_ in RB, TB, and TCB occurred in 50–60 cm layer and in 40–50 cm layer in BRB profile (Table 3).

The hot concentrated HCl extractable P_i_ (C.HCl-P_i_) and Residual-P were ranked as TCB (78.0 mg kg^−1^, 11.9%, and 47.1 mg kg^−1^, 7.2%) > TB (73.8 mg kg^−1^, 11.6%, and 46.9 mg kg^−1^, 7.4%) ≫ RB (61.6 mg kg^−1^, 9.5%, and 42.0 mg kg^−1^, 6.5%) > BRB (45.0 mg kg^−1^, 7.4%, and 36.9 mg kg^−1^, 6.0%) (Table 2 and Figure 2). Meanwhile, the content of the hot concentrated HCl extractable P_o_ (C.HCl-P_o_, mean: 3.4–4.9%), which was the lowest fraction of total extracted P_o_ in all the P forms, was ranked as BRB (30.1 mg kg^−1^, 4.9%) > TCB (28.6 mg kg^−1^, 4.3%) ≫ TB (22.7 mg kg^−1^, 3.6%) > RB (22.0 mg kg^−1^, 3.4%) (Table 2 and Figure 2). Meanwhile, there were no significant vertical differences with respect to the soil C.HCl-P_i_/P_o_ and Residual-P profiles (Figure 3).

### 3.3. Phosphorus Forms Related to Soil Geochemical Properties

The correlation analysis results showed that the Resin-P, Bicarb-P_i_, OH-P_i_, and C.HCl-P_i_ were strongly positively correlated with salinity (*r* = 0.76, 0.69, 0.61, and 0.64, resp.), TOC (*r* = 0.63, 0.65, 0.62, and 0.58, resp.), Ca (*r* = 0.75, 0.74, 0.60, and 0.72, resp.), Al (*r* = 0.75, 0.71, 0.54, and 0.73, resp.), and Fe (*r* = 0.65, 0.63, 0.48, and 0.63, resp.) but negatively correlated with pH (*r* = 0.48, 0.45, 0.60, and 0.50, resp.) (Table 4). The availability of P in most ecosystems depends on soil properties that regulate P availability, such as mineralogy of the parent material, leaching rates, and soil texture [27]. Part of the reason for the observed correlations might be that, in alkaline soils, P fixation which is biologically unavailable in many wetlands is governed by the activities of Ca and Mg [28–30]. We defined that forms of Bicarb-P_i_ and NaOH-P_i_ in soils represent a continuum of Fe- and Al-associated P extractable with high pH (8.7–9.2). Correlation and regression analyses showed that the amorphous and free Fe/Al oxides, in the newly formed wetland soils, were the crucial chemical factors ascribed to the soil P retention and release capacity [20].

Although the dilute extractable inorganic P has been clearly defined as Ca-associated P, D.HCl-P_i_ was not correlated with soil geochemical properties in our study (Table 4). Meanwhile, no significant correlations were found between organic P fractions (Bicarb-P_o_, NaOH-P_o_, and C.HCl-P_o_) and soil geochemical properties. All of the above results indicated that the there were actually links between P forms in estuarine coastal soils and the intensity of plant salt tolerance. This appearance was attributed to the fact that the formation of newborn coastal wetland in the YRD is only 35 years (1976–2011). Meanwhile, the soils in the wetland were formed from sediment eroded from the upland soils on the Loess Plateau and deposited in the Yellow River estuary. Deposition is usually episodic rather than smoothly continuous and this newborn wetland is still also influenced by water coming from the Yellow River and ocean tide [11, 13].

### 3.4. Vegetation Effects on the P Forms for Tidal River Network Region

Plants growing on tidal wetland are exposed to a number of extreme conditions, such as high wind velocities, drastic temperature fluctuations, high potential evapotranspiration, salt spray, low levels of soil nutrients, and burial in sediment [31, 32]. Vegetation has been proven to improve the soil P dynamics and availability [33–35]. Our results showed some clear differences between RB covered by* Phragmites australis* and TB covered by* Suaeda heteroptera *Kitag. (Table 2). The mean content of Resin-P, C.HCl-P_i_, Residual-P, and total inorganic P of TB soils (14.6 mg kg^−1^, 73.8 mg kg^−1^, 46.9 mg kg^−1^, and 570.2 mg kg^−1^, resp.) was higher than those of RB soils (11.0 mg kg^−1^, 61.6 mg kg^−1^, 42.0 mg kg^−1^, and 542.4 mg kg^−1^, resp.), while the mean content of Bicarb-P_o_, NaOH-P_o_, and total organic P of TB soils (17.7 mg kg^−1^, 25.7 mg kg^−1^, and 66.1 mg kg^−1^, resp.) was less than those of RB soils (40.8 mg kg^−1^, 43.9 mg kg^−1^, and 106.7 mg kg^−1^, resp.). Furthermore, there was no significant difference in mean content of Bicarb-P_i_, NaOH-P_i_, D.HCl-P_i_, C.HCl-P_o_, and total P of TB and RB. Vegetation cover also influenced the correlation coefficients between above P_i_ fractions and soil geochemical properties in the tidal river network region. The coefficients of RB covered by* Phragmites australis* and TB covered by* Suaeda heteroptera *Kitag. were higher than those of BRB and TCB with no vegetation cover. The results support the view that the soil P content varies with vegetation covers and have actually indicated links between the P forms in tidal wetland soils and the intensity of plant salt tolerance.

### 3.5. Hydrologic Disturbance Effects on the P Forms for Tidal River Network Region

Disturbance and stress are not uniformly high within coastal systems but exhibit strong gradients in physical disturbance, water availability, soil pH, nutrient level, and salinity [36]. Hydrology has been shown to influence a wetland soil ability to sorb [37, 38]. Berretta and Sansalone investigated the transport and partitioning of P to particulate matter fractions in runoff from a landscaped and biogenically loaded car park in Gainesville, FL (GNV), and found that P is predominantly bound to particulate matter (PM) fractions and the transport of each of PM and P fractions was influenced by separate hydrologic parameters [39]. Salinity was found to account for a significant proportion of variance in the phosphorus sorption index data [40]. Li et al. [15] simulated the effects of water level and salinity on nitrogen and phosphorus in salt marsh soils of the Yellow River Delta. Results showed that total nitrogen and total phosphorus contents accumulated in surface soils in each sampling date and that they are significantly affected by soil organic matter and salinity. Under such tidal-river conditions, water and salt of soil play a dominant role in the geochemical property in the area. In present research, it is also shown that SM and salinity significantly changed the phosphorus fractions in soils. Generally, the mean Resin-P, Bicarb-P_i_/P_o_, NaOH-P_i_/P_o_, C.HCl-P_i_, Residual-P, and total P_i_/P_t_ concentrations were TCB (seawater mainly influenced soil, higher value of SM and salinity) ≫ BRB (freshwater mainly influenced soil, lower value of SM and salinity) and there was no significant difference found in mean content of D.HCl-P_i_, C.HCl-P_o_, and total P_o_ (Table 4). The results also support the view that the soil P availability can increase by increasing salinity [41–43].

## 4. Conclusions

Our results proved that the modified Hedley fraction was effective in differentiating inorganic and organic P forms in this tidal river network region. The phosphorus in the investigated region exists in many complex chemical forms, which differ markedly in profile distribution in the four sampling plots. The P_t_ ranged from 612.1 to 657.8 mg kg^−1^. D.HCl-P_i_ was the predominant form of total extracted P_i_ and NaOH-P_o_ was the predominant form of total extracted P_o_, while Bicarb-P_i_ and C.HCl-P_o_ were the lowest fractions of total extracted P_i_ and P_o_, respectively, in all the P forms. The Pearson correlation matrix showed that Resin-P, Bicarb-P_i_, NaOH-P_i_, and C.HCl-P_i_ were strongly positively correlated with soil salinity, TOC, Ca, Al, and Fe, while all forms of organic P (Bicarb-P_o_, NaOH-P_o_, and C.HCl-P_o_) did not show any significant correlation with geochemical properties. Duncan multiple-range test indicated the P forms and distribution heterogeneity in the profiles can be attributed to the influences of different vegetation cover and hydrologic disturbance. The estimated distribution of different soil P fractions presented in the tidal river network region will be useful for wetland evolution that includes P as a limiting element in biological production by providing initial estimates of the available soil P for plant uptake and microbial utilization. Meanwhile, further investigation should be made to explore P availability and transformation dynamics in the soil under hydrologic disturbance.

## Figures and Tables

**Table 1:** General characteristics of the soils in the tidal river network region.

Soil	Depth (cm)	pH	SM (%)	Salinity (%)	TOC (%)	Ca (%)	Al (%)	Fe (%)
BRB	0–10	8.9 (0.08)	21.4 (1.40)	2.2 (1.22)	0.6 (0.25)	4.5 (0.86)	6.2 (0.70)	3.2 (0.52)
	10–20	9.1 (0.05)	21.1 (0.19)	0.7 (0.22)	0.5 (0.13)	4.3 (0.31)	6.3 (0.33)	2.8 (0.22)
	20–30	9.1 (0.12)	21.9 (0.40)	0.7 (0.37)	0.4 (0.11)	4.1 (0.06)	6.0 (0.10)	2.6 (0.08)
	30–40	9.2 (0.07)	22.6 (0.36)	0.5 (0.24)	0.3 (0.05)	4.0 (0.05)	5.8 (0.14)	2.5 (0.10)
	40–50	9.1 (0.10)	23.9 (0.49)	0.5 (0.07)	0.3 (0.06)	3.8 (0.38)	5.7 (0.32)	2.4 (0.09)
	50–60	9.2 (0.07)	24.5 (0.45)	0.4 (0.03)	0.3 (0.09)	3.7 (0.39)	5.6 (0.35)	2.5 (0.05)

RB	0–10	8.8 (0.28)	21.0 (1.83)	4.4 (4.32)	1.2 (0.18)	4.9 (0.50)	6.7 (0.46)	3.2 (0.46)
	10–20	8.8 (0.32)	20.7 (0.71)	4.7 (4.65)	0.7 (0.51)	4.7 (1.26)	6.5 (0.95)	3.0 (0.95)
	20–30	8.7 (0.24)	21.3 (0.83)	4.9 (3.92)	0.7 (0.19)	4.4 (0.53)	6.3 (0.36)	2.8 (0.42)
	30–40	8.8 (0.17)	21.9 (0.86)	3.9 (2.48)	0.4 (0.13)	4.2 (0.13)	6.0 (0.19)	2.6 (0.15)
	40–50	8.8 (0.14)	22.2 (0.70)	4.9 (2.51)	0.9 (0.66)	5.2 (1.54)	6.8 (0.94)	3.2 (0.82)
	50–60	8.9 (0.10)	22.3 (0.28)	4.4 (2.39)	0.7 (0.26)	5.3 (1.48)	6.8 (0.90)	3.2 (0.79)

TB	0–10	8.7 (0.05)	21.3 (1.19)	20.3 (1.12)	1.3 (0.11)	5.4 (0.20)	6.8 (0.24)	3.5 (0.06)
	10–20	8.9 (0.13)	21.7 (0.88)	9.6 (0.81)	1.0 (0.36)	5.6 (0.51)	6.9 (0.20)	3.7 (0.57)
	20–30	8.8 (0.06)	22.4 (0.96)	9.1 (0.17)	0.7 (0.12)	4.9 (0.49)	6.4 (0.31)	3.1 (0.45)
	30–40	8.8 (0.10)	23.0 (0.79)	9.4 (2.72)	0.7 (0.12)	4.9 (0.55)	6.5 (0.61)	3.2 (0.40)
	40–50	8.9 (0.16)	23.3 (0.58)	8.8 (2.14)	0.8 (0.32)	5.4 (0.74)	6.9 (0.55)	3.4 (0.57)
	50–60	8.9 (0.08)	24.4 (0.38)	7.9 (0.59)	0.9 (0.53)	5.0 (0.22)	6.6 (0.14)	3.2 (0.12)

TCB	0–10	8.7 (0.04)	20.9 (0.16)	22.2 (1.41)	1.0 (0.19)	6.1 (0.50)	7.5 (0.21)	3.1 (1.23)
	10–20	8.8 (0.10)	21.5 (0.56)	11.7 (1.50)	1.2 (0.13)	6.2 (0.41)	7.6 (0.29)	4.1 (0.36)
	20–30	8.9 (0.03)	22.2 (0.74)	9.5 (0.98)	1.1 (0.39)	5.6 (0.74)	7.2 (0.64)	3.6 (0.54)
	30–40	8.8 (0.10)	24.0 (0.32)	14.7 (5.90)	0.9 (0.23)	5.9 (0.56)	7.4 (0.53)	3.9 (0.36)
	40–50	8.9 (0.12)	24.6 (0.47)	9.6 (1.01)	0.9 (0.17)	5.6 (0.51)	7.2 (0.53)	3.6 (0.45)
	50–60	8.8 (0.06)	25.1 (0.63)	10.0 (1.95)	0.8 (0.22)	5.5 (0.92)	7.2 (0.78)	3.6 (0.66)

**Table 2:** Mean soil phosphorus concentrations by fraction in tidal river network region soils listed as mg P kg^−1^ soil. Means were averaged from 18 soil samples in each type. Duncan multiple-range test was conducted with 17 degrees of freedom. The signification of different forms of phosphorus was described in [8].

P extract	BRB	RB	TB	TCB	Geochemical significance	Ecological significance
Resin-P	9.6^c^	11.0^c^	14.6^b^	16.6^a^	Nonoccluded, rapid turnover	Plant available
Bicarb-P_i_	4.2^c^	4.9^bc^	5.8^b^	7.1^a^		Easily plant available,
Bicarb-P_o_	30.0^b^	40.8^a^	17.7^c^	37.6^a^		Easily mineralized
NaOH-P_i_	4.0^b^	5.4^a^	5.7^a^	6.2^a^	Nonoccluded, slow turnover	Lesser plant available
NaOH-P_o_	32.0^b^	43.9^a^	25.7^bc^	22.1^c^		Not directly plant available
D.HCl-P_i_	420.2^a^	417.4^a^	423.5^a^	414.6^a^	Occluded, slow turnover	
C.HCl-P_i_	45.0^c^	61.6^b^	73.8^a^	78.0^a^		
C.HCl-P_o_	30.1^a^	22.0^c^	22.7^bc^	28.6^ab^		
Residual-P	36.9^c^	42.0^b^	46.9^a^	47.1^a^		
P_i_	520.0^c^	542.4^b^	570.2^a^	569.5^a^		
P_o_	92.1^b^	106.7^a^	66.1^c^	88.2^b^		
P_t_	612.1^c^	649.2^ab^	636.3^b^	657.8^a^		

The a, b, c represent significant differences between means for groups, while means for groups in homogeneous subsets are displayed as ab and bc (*P* = 0.05, *n* = 18).

**Table 3:** Mean content (mg kg^−1^) of P forms of the tidal river network region soil profiles in the Yellow River Delta estuary.

Soil	Depth (cm)	Resin-P	Bicarb-P_i_	NaOH-P_i_	D.HCl-P_i_	C.HCl-P_i_	Residual-P	Bicarb-P_o_	NaOH-P_o_	C.HCl-P_o_	P_i_	P_o_	P_t_
BRB	0–10	12.5	5.2	4.5	414.1	51.3	41.0	26.6	34.9	30.4	528.6	102.5	620.5
	10–20	11.3	4.3	4.8	418.8	50.0	37.1	26.5	32.6	27.9	526.2	96.2	613.3
	20–30	9.5	4.7	4.0	414.5	42.4	33.4	33.7	33.5	28.2	508.6	100.6	604.0
	30–40	9.1	4.2	3.6	417.7	44.5	35.4	29.6	37.2	33.9	514.5	102.2	615.1
	40–50	7.9	3.1	3.6	440.5	41.8	39.9	31.9	25.7	32.5	536.9	97.5	627.1
	50–60	7.1	3.6	3.8	415.5	40.8	34.3	31.8	28.3	27.6	505.1	94.4	592.9

RB	0–10	14.6	5.8	5.3	423.1	61.1	40.2	50.6	44.9	19.0	550.0	135.7	664.5
	10–20	10.5	5.3	5.4	394.1	65.9	44.5	39.8	46.5	23.8	525.7	130.8	635.8
	20–30	9.8	4.0	5.0	410.2	51.6	44.5	33.7	38.9	20.3	525.1	117.1	618.0
	30–40	10.1	4.4	5.2	418.2	60.4	44.9	42.1	44.4	19.7	543.1	131.4	649.3
	40–50	10.5	4.8	5.9	417.1	70.6	40.1	41.8	44.3	28.2	549.0	126.1	663.3
	50–60	10.7	5.4	5.7	441.6	60.2	38.2	37.0	44.4	21.0	561.8	119.5	664.1

TB	0–10	18.4	8.2	7.5	421.9	78.7	46.4	20.7	26.6	26.4	581.1	93.7	654.8
	10–20	15.6	6.5	5.9	420.6	71.8	46.7	16.8	27.2	21.6	567.1	90.7	632.7
	20–30	12.8	4.5	5.1	420.5	67.2	46.3	14.1	22.7	19.1	556.4	83.1	612.4
	30–40	14.2	5.1	5.2	424.8	70.7	50.4	18.7	25.9	20.1	570.4	95.0	635.1
	40–50	14.5	5.4	5.3	420.8	83.5	50.8	20.5	25.8	28.3	580.3	97.0	654.9
	50–60	12.1	5.0	5.2	432.4	70.6	40.6	15.2	26.0	20.7	565.8	81.8	627.8

TCB	0–10	18.8	9.7	7.7	419.6	82.4	44.3	40.1	24.3	29.7	582.5	108.6	676.5
	10–20	18.7	7.7	6.6	414.2	71.3	49.3	40.6	15.9	30.1	567.9	105.8	654.5
	20–30	15.4	6.5	5.4	416.7	75.9	50.9	36.5	24.0	22.5	570.8	111.4	653.8
	30–40	15.8	6.5	5.4	407.7	71.1	45.6	36.8	18.7	33.4	552.0	101.2	640.9
	40–50	16.0	6.4	5.6	407.6	83.2	47.0	37.5	23.9	29.7	565.8	108.4	656.9
	50–60	15.0	5.6	6.2	421.9	84.2	45.3	33.8	25.8	26.1	578.3	104.9	664.0

**Table 4:** Pearson correlation coefficients between P fractions and soil geochemical properties.

Sites		Pearson correlation								
		Resin-P	Bicarb-P_i_	NaOH-P_i_	D.HCl-P_i_	C.HCl-P_i_	Bicarb-P_o_	NaOH-P_o_	C.HCl-P_o_	Residual-P
BRB (*n* = 18)	pH	−0.66**								
	SM	−0.77**								
	Salinity	0.50*								
	TOC	0.59*	0.64**							
	Ca	0.65**				0.78**				
	Al	0.59*				0.74**				
	Fe	0.73**	0.56*			0.76**				

RB (*n* = 18)	pH			−0.66**				0.61**	−0.58*	−0.58*
	SM	−0.47*	−0.49**							
	Salinity		0.54**						0.55*	
	TOC	0.59*	0.75**	0.61**						
	Ca	0.48*	0.79**	0.76**		0.55**			0.48*	
	Al	0.54*	0.83**	0.73**		0.53**			0.49*	
	Fe	0.59*	0.86**	0.73**		0.51**			0.50*	

TB (*n* = 18)	pH									
	SM			−0.54**						
	Salinity	0.53*	0.60**	0.77**						
	TOC	0.45*	0.60**	0.66**						
	Ca	0.59**	0.71**			0.72**			0.50*	
	Al	0.59**	0.61**			0.82**	0.45*		0.51*	
	Fe	0.64**	0.80**			0.67**			0.46*	

TCB (*n* = 18)	pH			−0.50*						
	SM	−0.47*	−0.49**							
	Salinity		0.54**	0.48*						
	TOC									
	Ca	0.50*	0.79**		0.50*		0.51**	0.55*		
	Al	0.51*	0.83**		0.51*		0.57**	0.51*	0.55*	
	Fe									

All (*n* = 72)	pH	−0.48**	−0.45**	−0.60**		−0.50**				−0.41**
	SM							−0.28*		
	Salinity	0.76**	0.69**	0.61**		0.64**		−0.36**		0.43**
	TOC	0.63**	0.65**	0.62**		0.58**				0.34**
	Ca	0.75**	0.74**	0.60**		0.72**				0.36**
	Al	0.75**	0.71**	0.54**		0.73**				0.36**
	Fe	0.65**	0.63**	0.48**		0.63**				0.33**

*Significant at the 0.05 level (2-tailed).

**Significant at the 0.01 level (2-tailed).

## References

[1] Doering PH, Oviatt CA, Nowicki BL, Klos EG, Reed LW (1995). Phosphorus and nitrogen limitation of primary production in a simulated estuarine gradient. *Marine Ecology Progress Series*.

[2] Pierzynski GM, Vance GF, Sims JT (2005). *Soils and Environmental Quality*.

[3] Stevenson FJ, Cole MA (1999). *Cycles of Soils: Carbon, Nitrogen, Phosphorus, Sulfur, Micronutrients*.

[4] Levy ET, Schlesinger WH (1999). A comparison of fractionation methods for forms of phosphorus in soils. *Biogeochemistry*.

[5] Carter MR, Gregorich EG (2008). *Soil Sampling and Methods of Analysis*.

[6] Li M, Zhang J, Wang GQ, Yang HJ, Whelan MJ, White SM (2013). Organic phosphorus fractionation in wetland soil profiles by chemical extraction and phosphorus-31 nuclear magnetic resonance spectroscopy. *Applied Geochemistry*.

[7] Hedley MJ, Stewart JWB, Chauhan BS (1982). Changes in inorganic and organic soil phosphorus fractions induced by cultivation practices and by laboratory incubations. *Soil Science Society of America Journal*.

[8] Cross AF, Schlesinger WH (1995). A literature review and evaluation of the Hedley fractionation: applications to the biogeochemical cycle of soil phosphorus in natural ecosystems. *Geoderma*.

[9] Cross AF, Schlesinger WH (2001). Biological and geochemical controls on phosphorus fractions in semiarid soils. *Biogeochemistry*.

[10] Wang G-P, Zhai Z-L, Liu J-S, Wang J-D (2008). Forms and profile distribution of soil phosphorus in four wetlands across gradients of sand desertification in Northeast China. *Geoderma*.

[11] Ye Q, Chen S, Chen Q (2006). Spatial-temporal characteristics in landscape evolution of the Yellow River Delta during 1855–2000 and a way out for the Yellow River estuary. *Chinese Science Bulletin*.

[12] Milliman JD, Syvitski JPM (1992). Geomorphic/tectonic control of sediment discharge to the ocean: the importance of small mountainous rivers. *The Journal of Geology*.

[13] Yu J, Fu Y, Li Y (2011). Effects of water discharge and sediment load on evolution of modern Yellow River Delta, China, over the period from 1976 to 2009. *Biogeosciences*.

[14] Song C, Liu G (2008). Application of remote sensing detection and gis in analysis of vegetation pattern dynamics in the Yellow River Delta. *Chinese Journal of Population, Resources and Environment*.

[15] Li S-N, Wang G-X, Deng W, Hu Y-M, Hu W-W (2009). Influence of hydrology process on wetland landscape pattern: a case study in the Yellow River Delta. *Ecological Engineering*.

[16] Ouyang W, Wang X, Hao F, Srinivasan R (2009). Temporal-spatial dynamics of vegetation variation on non-point source nutrient pollution. *Ecological Modelling*.

[17] Cui B, Yang Q, Yang Z, Zhang K (2009). Evaluating the ecological performance of wetland restoration in the Yellow River Delta, China. *Ecological Engineering*.

[18] Fan H, Huang H, Zeng T (2006). Impacts of anthropogenic activity on the recent evolution of the Huanghe (Yellow) River delta. *Journal of Coastal Research*.

[19] Wang LL, Ye M, Li QS, Zou H, Zhou YS (2013). Phosphorus speciation in wetland sediments of Zhujiang (Pearl) River Estuary, China. *, Chinese Geographical Science*.

[20] Xu G, Shao HB, Sun JN, Chang SX (2012). Phosphorus fractions and profile distribution in newly formed wetland soils along a salinity gradient in the Yellow River Delta in China. *Journal of Plant Nutrition and Soil Science*.

[21] Pan G, Krom MD, Zhang MY (2013). Impact of suspended inorganic particles on phosphorus cycling in the Yellow River (China). *Environmental Science & Technology*.

[22] Sun JN, Xu G, Shao HB, Xu SH (2012). Potential retention and release capacity of phosphorus in the newly formed wetland soils from the yellow river delta, China. *Clean-Soil Air Water*.

[23] Murphy J, Riley JP (1962). A modified single solution method for the determination of phosphate in natural waters. *Analytica Chimica Acta*.

[24] Tiessen H, Moir JQ (2008). Characterization of available P by sequential extraction. *Soil Sampling and Methods of Analysis*.

[25] Yu J, Chen X, Sun Z (2010). The spatial distribution characteristics of soil nutrients in new-born coastal wetland in the Yellow River delta. *Acta Scientiae Circumstantiae*.

[26] Verma S, Subehia SK, Sharma SP (2005). Phosphorus fractions in an acid soil continuously fertilized with mineral and organic fertilizers. *Biology and Fertility of Soils*.

[27] Mishra A, Tripathi JK, Mehta P, Rajamani V (2013). Phosphorus distribution and fractionation during weathering of amphibolites and gneisses in different climatic setups of the Kaveri river catchment, India. *Applied Geochemistry*.

[28] Masto RE, Mahato M, Selvi VA, Ram LC (2013). The effect of fly ash application on phosphorus availability in an acid soil. *Energy Sources A: Recovery Utilization and Environmental Effects*.

[29] Paiva DM, Walk CL, McElroy AP (2013). Influence of dietary calcium level, calcium source, and phytase on bird performance and mineral digestibility during a natural necrotic enteritis episode. *Poultry Science*.

[30] Wadu MCWM, Michaelis VK, Kroeker S, Akinremi OO (2013). Exchangeable calcium/magnesium ratio affects phosphorus behavior in calcareous soils. *, Soil Science Society of America Journal*.

[31] Maun MA (1998). Adaptations of plants to burial in coastal sand dunes. *Canadian Journal of Botany*.

[32] Sun Z, Mou X, Lin G, Wang L, Song H, Jiang H (2010). Effects of sediment burial disturbance on seedling survival and growth of Suaeda salsa in the tidal wetland of the Yellow River estuary. *Plant and Soil*.

[33] Tuchman NC, Larkin DJ, Geddes P, Wildova R, Jankowski K, Goldberg DE (2009). Patterns of environmental change associated withTypha xglauca invasion in a Great Lakes coastal wetland. *Wetlands*.

[34] Boomer KMB, Bedford BL (2008). Groundwater-induced redox-gradients control soil properties and phosphorus availability across four headwater wetlands, New York, USA. *Biogeochemistry*.

[35] Bonanomi G, Rietkerk M, Dekker SC, Mazzoleni S (2008). Islands of fertility induce co-occurring negative and positive plant-soil feedbacks promoting coexistence. *Plant Ecology*.

[36] Balestri E, Lardicci C (2013). The impact of physical disturbance and increased sand burial on clonal growth and spatial colonization of Sporobolus virginicus in a coastal dune system. *PLoS ONE*.

[37] Axt JR, Walbridge MR (1999). Phosphate removal capacity of palustrine forested wetlands and adjacent uplands in Virginia. *Soil Science Society of America Journal*.

[38] Chambers RM, Odum WE (1990). Porewater oxidation, dissolved phosphate and the iron curtain: iron-phosphorus relations in tidal freshwater marshes. *Biogeochemistry*.

[39] Berretta C, Sansalone J (2011). Hydrologic transport and partitioning of phosphorus fractions. *Journal of Hydrology*.

[40] Bruland GL, DeMent G (2009). Phosphorus sorption dynamics of hawaii’s coastal wetlands. *Estuaries and Coasts*.

[41] Fox LE, Sager SL, Wofsy SC (1986). The chemical control of soluble phosphorus in the Amazon estuary. *Geochimica et Cosmochimica Acta*.

[42] House WA (1999). The physico-chemical conditions for the precipitation of phosphate with calcium. *Environmental Technology*.

[43] Sundareshwar PV, Morris JT (1999). Phosphorus sorption characteristics of intertidal marsh sediments along an estuarine salinity gradient. *Limnology and Oceanography*.

